# The Potential Morphological Stenosis Pattern of the Arcuate Foramen

**DOI:** 10.3390/diagnostics15101203

**Published:** 2025-05-09

**Authors:** Ioannis Paschopoulos, Maria Piagkou, George Triantafyllou, Panagiotis Papadopoulos-Manolarakis, Fabrice Duparc, Fotis Demetriou, George Tsakotos, Rǎzvan-Costin Tudose, Mugurel Constantin Rusu, Oana Daniela Toader

**Affiliations:** 1 Department of Anatomy, School of Medicine, Faculty of Health Sciences, National and Kapodistrian University of Athens, 11527 Athens, Greece; johnpascho@gmail.com (I.P.); georgerose406@gmail.com (G.T.); p.papado89@gmail.com (P.P.-M.); fotisdemetriou2000@gmail.com (F.D.); gtsakotos@gmail.com (G.T.); 2 Department of Neurosurgery, General Hospital of Nikaia-Piraeus, 18454 Nikaia, Greece; 3 Department of Anatomy, Faculty of Medicine-Pharmacy, University of Rouen-Normandy, 76821 Rouen, France; fabrice.duparc@univ-rouen.fr; 4 Division of Anatomy, Faculty of Dentistry, “Carol Davila” University of Medicine and Pharmacy, 020021 Bucharest, Romania; razvan-costin.tudose0721@stud.umfcd.ro (R.-C.T.); mugurel.rusu@umfcd.ro (M.C.R.); 5 Department 13 of Obstetrics, Gynecology and Neonatology, “Polizu” Clinical Hospital, “Carol Davila” University of Medicine and Pharmacy, 020021 Bucharest, Romania; oana.toader@umfcd.ro

**Keywords:** vertebral artery, anatomy, compression syndromes, variation

## Abstract

**Background**: The arcuate foramen (AF), an osseous foramen, is probably formatted from the ossification of the posterior atlanto-occipital membrane. When this morphologically ossified variant exists, it encloses the vertebral artery (VA) third segment (V3). This close relationship may cause compression to the VA with concomitant vertebrobasilar insufficiency, vertigo, headaches, or neck pain. In the published literature, no studies investigate the abovementioned potential compression pattern. The present study examines the AF ossification pattern (complete or partial type) and the variable VA diameter at the atlantal part (V3), concluding a potential risk for VA compression after correlating the relative diameters (AF and VA diameters). **Materials and Methods**: One hundred and fifty dried first cervical vertebrae (atlases) and one hundred fifty computed tomography (CT) scans were obtained for the present study. The presence of a complete or incomplete AF was evaluated, and when present, its diameter was measured. To correlate these findings with the vessel, 50 computed tomography angiographies (without AF presence) were obtained to measure the V3 segment diameter. **Results**: Out of the total 600 (*N* = 600) sides, 111 sides had incomplete AF (18.2%), and 67 sides had complete AF (11.1%). The AF mean diameter was 6.41 (1.12) mm. The diameter of the V3 segment ranged between 5.0 and 6.0 mm; therefore, three morphological stenosis patterns were identified. A low risk of compression (over 6.0 mm) was identified in 61.2% (*N* = 109 sides), a moderate risk (between 5.0–6.0 mm) was observed in 29.2% (*N* = 52 sides), and a high risk (under 5.0 mm) was recorded in 9.6% (*N* = 17 sides). There was no statistically significant correlation regarding sexes and age for the potential compression patterns. **Conclusions**: The present study revealed the morphological stenosis pattern of the AF to the V3 segment. The variation had a high risk of compression to the vessel in 9.6% of sides, indicating that it is not infrequent. Knowledge of these details is essential for clinicians when investigating vertebrobasilar insufficiency.

## 1. Introduction

The vertebral artery (VA) is a major neck artery that supplies blood to the brain’s upper spinal cord, brainstem, cerebellum, and posterior part. Typically, it originates from the first part of the subclavian artery and extends superiorly and posteriorly, passing between the longus colli and the anterior scalene muscles. The two VAs penetrate deeply into the transverse process at the sixth cervical vertebra level and continue into each cervical vertebra’s transverse foramen (TF). After passing through the TF of the atlas (C1), the VAs transverse the C1 posterior arch and continue through the suboccipital triangle before entering the foramen magnum. Their confluence forms the basilar artery. The basilar and internal carotid arteries give off several communicating branches that anastomose at the base of the brain, creating the cerebral arterial circle, which connects the anterior and posterior circulations [1].

**Figure 1:**
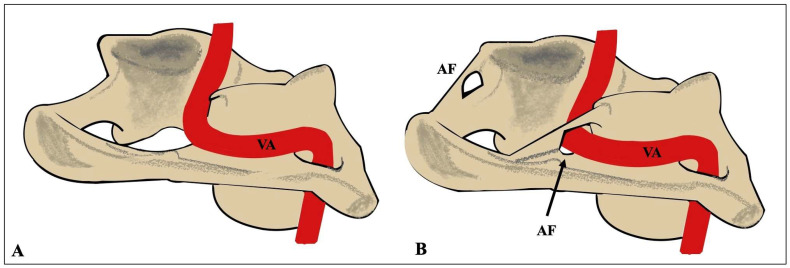
Schematic representation of the typical course of the vertebral artery (VA) at the level of the first cervical vertebra (**A**) and the course when the arcuate foramen (AF) is present (**B**).

On their path, the VAs transverse the atlas posterior arch, where the arcuate foramen (AF) may form. The AF is a bony foramen likely created from the ossification of the posterior atlanto-occipital membrane [2]. The literature also refers to the ponticulus posticus, pons ponticus, foramen sagittale, retroarticular VA ring, atlas bridging, or the Kimmerle anomaly [3]. The existence of this ossified variant may lead to the enclosure of the VA’s third segment (V3) (Figure 1). This variation is categorized into partial and complete forms based on the percentage of existing ossification. Numerous studies have explored the relationship between the AF and the VA, demonstrating an association between the AF and conditions such as cervical pain, headaches, hearing loss, vertebrobasilar insufficiency, vertigo, and neck pain attributed to vessel compression [2,4]. Gul and Atik [4] identified that the VA diameter was reduced when the AF was present, which could be a potential cause for vertebrobasilar insufficiency.

It is also important to highlight that the C1 vertebra exhibits considerable morphological variability, and additional variations can be identified in the area. The AF is one morphological variant surrounding the VA at this level. Another ossified (lateral) variant in the atlas region is a lateral osseous bridge, the lateral foramen (LF), which forms when the lateral mass extends sideways to the TF [5]. Nevertheless, the coexistence of AF and LF creates the posterolateral foramen (PLF) [5]. Pekala et al. [5] estimated the pooled prevalence of the complete LF to be 2.6% and the complete PLF to be 1.2%, depicting that these morphological variants are significantly rarer than the AF. Therefore, the current anatomical imaging study will focus on the AF presence and possible compression to the VA due to its significantly higher frequency.

In the published literature, they are missing studies examine the potential compression patterns of the AF to the VA, akin to other regions of the human body [6,7]. Given the significant clinical implications of VA compression or impingement caused by variably ossified AF, this study seeks to assess the diameter of the AF and its potential to compress the VA.

## 2. Materials and Methods

**Figure 2:**
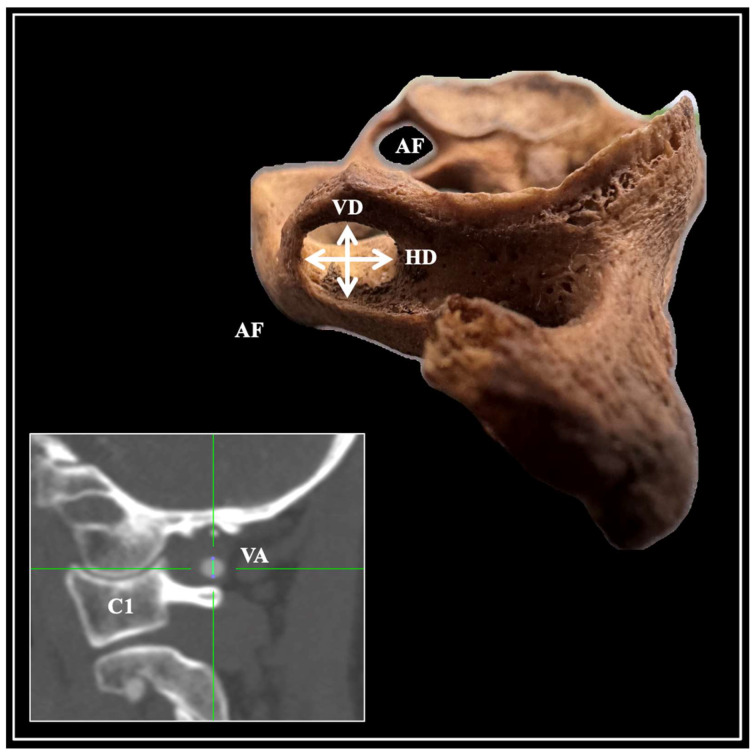
The morphometric measurements obtained for this study include the arcuate foramen (AF) diameters on a dried atlas vertebra and the vertebral artery (VA) diameter on the atlas VA groove, free of an AF presence. VD is the vertical diameter, and HD is the horizontal diameter.

The sample comprised dried C1 vertebrae, computed tomography scans (CTs), and CT angiographies (CTAs). The presence of AF (incomplete or complete) was determined at the posterior arch of the C1 vertebra, similar to previous studies, and it was differentiated from the LF [5]. At present, the AF’s vertical and horizontal diameters were measured (Figure 2). A total of 300 C1 vertebrae were evaluated (600 observations).

One hundred fifty (150) dried adult C1 vertebrae were obtained from the osteological collection of the Anatomy Department, School of Medicine, National and Kapodistrian University of Athens, and they were investigated for AF presence and its vertical and horizontal diameters (VD and HD). The exact ages, sexes, and chronology of the sample were unknown. The specimens were derived from the “Body Donation Program” of the University (affiliation 1) with informed consent of the cadavers before death [8].

Additionally, 150 CT scans of skulls and cervical vertebrae were obtained from the General Hospital of Nikaia-Piraeus (affiliation 2), following ethical approval from the responsible authorities (number: 56,485/date: 13 November 2024). The scans were from 85 female and 65 male patients free of disease, with a mean age of 47.06 years (ranging from 20 to 79).

To correlate the osteological findings with the vessel, the VA diameter was measured at the VA groove (VAG) in patients without AF to avoid any potential effects of variation on the vessel. The VA was evaluated in 50 CTAs of 25 female and 25 male patients without pathological conditions that could distort the regional anatomy (Figure 2).

The investigation was performed using a 128 multi-slice CT scanner and documented with Horos software version 3.3.6 (Horos Project, New York, NY, USA). Similar to previous studies, evidence was obtained from the multiplanar reconstruction of the axial, coronal, and sagittal slices, along with their three-dimensional volume reconstruction [9,10]. In the dry sample, the cases were documented and measured with the use of the ImageJ software version 1.54p (National Institutes of Health, Bethesda, MD, USA).

Statistical analysis was performed with IBM Statistics for MacOS IBM SPSS Statistics for MacOS, Version 29 (IBM Corp., Armonk, New York, NY, USA). Nominal data between unpaired observations were compared using the Chi-square test, while McNemar’s test was applied for paired observations. Normality was assessed with the Shapiro–Wilk test. Continuous variables were analyzed based on measurement type; unpaired measurements were evaluated with an independent *t*-test if normality was met; otherwise, the Mann–Whitney U test was used. A paired *t*-test was employed for paired measurements when normality was satisfied. Mean comparisons across more than two groups involved one-way ANOVA if normal distribution was present; if not, the Kruskal–Wallis test was used. Unless otherwise specified, results are presented as mean and standard deviation (SD). A *p*-value less than 0.05 was considered statistically significant.

## 3. Results

**Figure 3:**
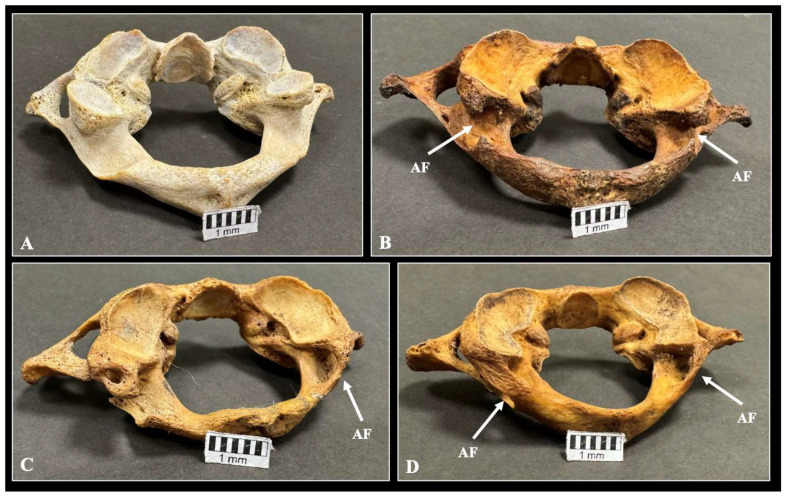
The variants of the arcuate foramen (AF) are illustrated in osteological specimens: (**A**) bilateral absence (symmetry), (**B**) bilateral incomplete morphology (symmetry), (**C**) unilateral complete morphology (asymmetry), and (**D**) bilateral complete morphology (symmetry).

**Figure 4:**
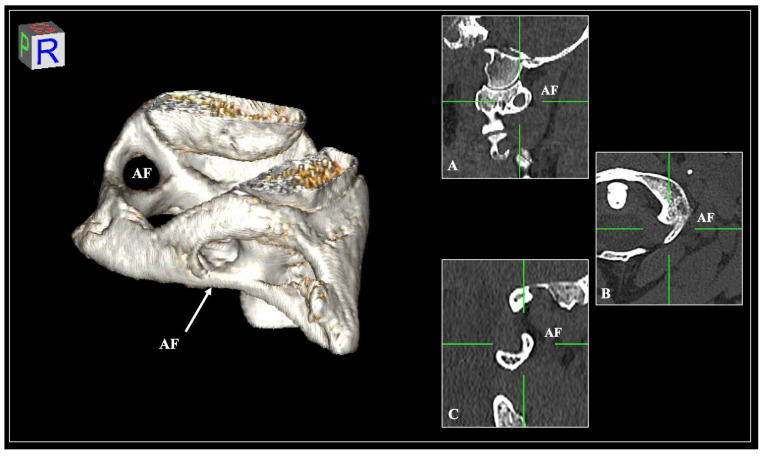
The arcuate foramen (AF) is depicted in a computed tomography scan. The AF is well-represented in sagittal slices (**A**) but not in axial and coronal slices (**B**,**C**).

Out of a total of 600 sides, the AF was observed in 178 sides (*N* = 178/600, 29.6%), unilaterally in 120 vertebrae (*N* = 120/300, 40%) and bilaterally in 29 vertebrae (*N* = 29/300, 9.6%). The AF of incomplete type was recorded in 111 sides (*N* = 111/600, 18.5%), and of complete type was noted in 67 sides (*N* = 67/600, 11.2%). The types of AF are depicted in the osteological specimens in Figure 3 and the CT scans in Figure 4. The distribution of sides and sexes is summarized in Table 1. No significant differences were found. Moreover, when comparing the cases between the CTs and dry atlases, there was no statistically significant difference (*p* = 0.874).

The AF’s mean maximum vertical diameter (VD) was 6.41 mm (*N* = 149 AFs), ranging from 2.94 mm to 9.81 mm. The AF’s mean maximum horizontal or transverse diameter (HD or TD) was 6.20 mm (*N* = 149 AFs), ranging from 4.05 mm to 8.69 mm. The morphometric measurements categorized by sides and sexes are summarized in Table 2. The VA TD varied between 5.0 mm (minimum) and 6.0 mm (maximum). No significant association was found between sides and sexes. When comparing the diameters obtained from the CT scans and the osteological specimens, no statistically significant differences were obtained (*p* = 0.547).

After calculating the AF morphometric measurements and correlating them with the VA diameter, the VA potential compression due to the presence of the AF morphological variant was determined and summarized in Table 3 (for the VD) and Table 4 (for the HD). Three possible compression patterns were observed based on the minimum (5.0 mm) and maximum (6.0 mm) diameters of the VA. A low risk of stenosis was considered when the AF had a VD over 6.0 mm; this type was identified in 109 sides (*N* = 109/178, 61.2%). An intermediate risk of compression was recorded when the AF VD ranged between 5.0 mm and 6.0 mm, observed in 52 sides (*N* = 52/178, 29.2%). A high risk of compression was noted when the AF had a VD of less than 5.0 mm, identified in 17 sides (*N* = 17/178, 9.6%). The distribution based on sides, sexes, and AF morphology is summarized in Table 3.

A low risk of stenosis was considered when the AF had an HD over 6.0 mm; this type was identified in 98 sides (*N* = 98/178, 55.1%). An intermediate risk of compression was recorded when the AF HD ranged between 5.0 mm and 6.0 mm, observed in 58 sides (*N* = 58/178, 32.6%). A high risk of compression was noted when the AF had an HD of less than 5.0 mm, identified in 22 sides (*N* = 22/178, 12.4%). The distribution based on sides, sexes, and AF morphology is summarized in Table 4. A slightly higher prevalence of high risk of compression was identified between the HD (12.4%) versus the VD (9.6%), which was not statistically significant.

## 4. Discussion

In the present study, we examined the presence of the AF and its morphometric characteristics, precisely the AF diameter, to explore the potential compression of the VA. Our findings indicate that 9.6% of the AF exhibited a significant risk of arterial compression, as their VD was below the mean diameter of the VA, while 12.4% of AF had a considerable risk of compression, as their HD was below the mean diameter of the VA. This method was not previously performed for the AF-VA diameter relationship; however, in an analog modality, it was investigated for the internal carotid artery (ICA) compression at the caroticoclinoid foramen [7] and the suprascapular nerve stenosis at the suprascapular notch [6]. The morphological variability and clinical implications of AF are discussed further.

The present study identified the presence of AF in 178 out of 600 examined sides (29.6%). The incomplete type was observed in 111 sides (18.5%), while the complete type was noted in 67 sides (11.2%). These findings are slightly higher than those of the meta-analysis conducted by Pekala et al. [11], which reported a pooled prevalence of 13.6% for the incomplete type and 9.1% for the complete type. When evaluated by study type, CT-based studies showed a prevalence of 14.9% for the AF incomplete type and 10.8% for the complete type. In comparison, cadaveric studies reported a prevalence of 15.1% for the incomplete AF and 9.7% for the complete type. These results differ slightly from those of our research. It is important to mention that we did not observe statistically significant differences when comparing the two methods on our sample. Thus, we could assume that CT scans (with a slice thickness of maximum 0.8 mm) are reliable to identify the AF. However, the meta-analysis highlights the significant variability observed among different geographical subgroups. Specifically, the incomplete type of AF is most common among African populations (30.2%), while it is least common among Turkish populations (9.6%). The complete type is most prevalent among North American populations (11.3%) and is notably rare among Asian populations, particularly Chinese (4.4%) [11]. The prevalence of AF, complete or incomplete, among the published studies, can show an extravagant difference. Studies show its prevalence can vary significantly between different populations (Table 5). The aforementioned geographical differences suggest that genetic factors might contribute to the AF formation, either complete or incomplete. Specifically, the family study conducted by Saunders and Popovic [12] showed significant correlations in AF formation among parents, offspring, and siblings. These results suggest that AF may be a hereditary variation.

It remains uncertain whether age-related degenerative changes can lead to AF (either complete or partial) formation. Specifically, Paraskevas et al. [33] found a transformation of an incomplete AF to complete due to calcification but could not find evidence to support it. These results correlate with other studies that found no relationship between the formation of AF (complete or incomplete) and age [33]. The statements above regarding the relationship between AF formation and age or genetic factors suggest that its formation mechanism is not yet clearly understood [33].

The prevalence of complete and incomplete (partial) AF types varies significantly among ethnic groups. However, notable differences in prevalence between sides and sexes differ across studies. Regarding those results, Natsis et al. [32] found a prevalence of 18.03% for the AF partial type and 15.98% for the AF complete type, significantly close to ours. They also did not find a significant difference between the sides and sexes, which aligns with our results. Opposing those results is the meta-analysis by Pekala et al. [11], who found a slight male dominance for the AF complete type and a female dominance for the incomplete type. No significant difference was found between the sides.

In their study, Vanek et al. [32] found that the AF unilateral prevalence was 7.6%, while the bilateral prevalence was 6.7%, similar to our study’s results. Specifically, we found the AF bilateral prevalence to be 9.6% and the unilateral prevalence to be 40%. Regarding the same results, Gul and Atik [4] found a total prevalence of AF at 21.1%, with a unilateral prevalence of 11.2% and a bilateral prevalence of 9.8%.

Regarding the AF morphometry in our study, its mean VD was measured at 6.41 mm, ranging between 2.94 mm and 9.81 mm. The mean HD was measured at 6.20 mm, ranging from 4.05 mm to 8.69 mm. Given the variability in VA diameters, which typically range between 5.00 mm and 6.00 mm, the potential risk of compression varies accordingly. Gul and Atik [4] found the AF mean diameter to be 6.40 mm. Following the same results, Pekala et al. [11], in their meta-analysis, found the AF to have a mean HD of 5.65 mm and mean VD of 5.16 mm. Those results derive from the meta-analysis of eight cadaveric studies involving 125 subjects [11].

The AF’s clinical significance pertains to its potential compression or impingement of the VA. Notably, the existence of an AF in patients frequently coincides with the VA stretching during rotation at the C1–C2 level. As the artery traverses the AF on the side contralateral to the direction of head rotation, it becomes elongated and narrowed, impeding blood flow due to compression against the atlas lateral mass. This impingement can lead to headaches, vertigo, hearing loss, neuralgia, neck pain, loss of consciousness, and vertebrobasilar ischemia [2]. Pekala et al. [34] showed that VA compression can result in migraines and cervicogenic headaches with no significant association between one- or two-sided AF and the presence of headaches.

Pekala et al. [11], in their meta-analysis, emphasized that neurosurgeons should, above all others, be aware of a complete AF observation, particularly during screw placement on the contralateral side of the atlas, as there is a 53.1% probability that an AF is also present there. The presence of the AF can lead to trauma or compression of the VA, resulting in various pathological outcomes. It is evident that the complete AF forms a ring around the VA, and its area has been found to be smaller than the artery’s diameter on certain occasions. Thus, the VA can be compressed, significantly increasing the likelihood of pathological symptomatology. Many studies have reported an association between a complete AF and neurological symptoms, such as vertigo, migraine, and Barre–Lieou syndrome. Furthermore, a significant improvement in symptoms has been observed following the surgical removal of the bony bridge surrounding the VA [34].

We must acknowledge a few limitations of the current study. The samples were derived from a specific geographic region (Athens, Greece), and the sample was deemed adequate for our analysis (*n* = 300 atlas vertebrae). To unify our results, the AF were classified based on their diameter, regardless of their type (complete or incomplete). Lastly, it is essential to mention that the cD the VA from CTAs) were conducted to correlate the AF with VA diameters free from pathological conditions. Future studies investigating both clinical symptoms would be even more advantageous.

## 5. Conclusions

Three morphological stenosis patterns were documented based on the VA’s diameter at the AF’s location. The most significant finding of the current study indicates that 9.6% (VD) and 12.2% (HD) of AF exhibit a heightened risk of compression to the VA, specifically those with a diameter of less than 5.00 mm. Future research employing this methodology will advance our understanding of potential variations related to sex, side, or nationality and their possible clinical implications, such as clinical signs and symptoms. Given the variable prevalence of the AF and the different VA diameter between populations, researchers could explore the morphological stenosis patterns to identify possible differences between ethnic groups. Knowledge of AF variants is crucial for radiologists and neurosurgeons during interventional procedures.

## Figures and Tables

**Table 1:** The arcuate foramen (AF) presence regarding its morphology, by side and sex.

Presence of AF	Total *n* (%)	Left *n* (%)	Right *n* (%)	*p*-Value	Female *n* (%)	Male *n* (%)	*p*-Value
Incomplete type	111 (18.5)	58 (19.3)	53 (17.6)	0.899	57 (33.5)	37 (28.4)	0.238
Complete type	67 (11.2)	33 (11)	34 (11.3)	0.897	14 (8.2)	19 (14.6)	0.133

**Table 2:** The arcuate foramen (AF) diameters regarding sides and sexes. The results are presented as mean (standard deviation, SD).

AF Morphometry (Diameters)	Total Mean (SD)	Left Mean (SD)	Right Mean (SD)	*p*-Value	Female Mean (SD)	Male Mean (SD)	*p*-Value
Vertical (VD)-*n* = 149 AFs	6.41 (1.12)	6.42 (1.09)	6.40 (1.15)	0.911	6.56 (1.01)	6.46 (1.03)	0.591
Horizontal (HD)-*n* = 149 AFs	6.20 (1.02)	6.33 (1.04)	6.06 (0.98)	0.126	6.12 (1.02)	6.30 (1.02)	0.320

**Table 3:** The morphological stenosis pattern of the arcuate foramen (AF) is based on its vertical diameter compared to the vertebral artery (VA) diameter.

Parameters	Possible Risk Calculation		
	Low Risk (>6.0 mm)	Intermediate Risk (5.0–6.0 mm)	High Risk (<5.0 mm)
Total-*n* (%)	109 (61.2%)	52 (29.2%)	17 (9.6%)
By side			
Left-*n* (%)	58 (32.5%)	25 (14.04%)	8 (4.4%)
Right-*n* (%)	51 (28.6%)	27 (15.16%)	9 (5.0%)
*p*-value	0.780		
By sex			
Female-*n* (%)	48 (26.9%)	19 (10.6%)	4 (2.2%)
Male-*n* (%)	36 (20.2%)	16 (8.9%)	4 (2.2%)
*p*-value	0.124		
By the AF morphology presence			
AF incomplete type-*n* (%)	74 (41.57%)	29 (16.2%)	8 (4.4%)
AF complete type-*n* (%)	35 (19.66%)	23 (12.9%)	9 (5.0%)
*p*-value	0.130		

**Table 4:** The morphological stenosis pattern of the arcuate foramen (AF) is based on its horizontal diameter compared to the vertebral artery (VA) diameter.

Parameters	Possible Risk Calculation		
	Low Risk (>6.0 mm)	Intermediate Risk (5.0–6.0 mm)	High Risk (<5.0 mm)
Total-*n* (%)	98 (55.1%)	58 (32.6%)	22 (12.4%)
By side			
Left-*n* (%)	52 (29.2%)	28 (15.7%)	11 (6.2%)
Right-*n* (%)	46 (25.8%)	30 (16.9%)	11 (6.2%)
*p*-value	0.841		
By sex			
Female-*n* (%)	38 (21.3%)	24 (13.5%)	9 (5.1%)
Male-*n* (%)	35 (19.7%)	17 (9.6%)	4 (2.2%)
*p*-value	0.485		
By the AF morphology presence			
AF incomplete type-*n* (%)	66 (37.1%)	30 (16.9%)	15 (8.4%)
AF complete type-*n* (%)	32 (18.0%)	28 (15.7%)	7 (3.9%)
*p*-value	0.125		

**Table 5:** The morphological variability of the arcuate foramen (AF) in the current published literature. NR, not reported.

Author	Year	Country	No. of Subjects	AF Frequency	
				Complete%	Incomplete%
Zaborowski [13]	1975	Poland	4046	8.7	2.9
Sato and Noriyasu [14]	1978	Japan	1428	5.5	NR
Stubbs et al. [15]	1991	USA	1000	13.5	5.2
Mitchel [16]	1998	South Africa	1354	13.3	NR
Wysocki et al. [17]	2003	Poland	95	13.7	17.9
Beck et al. [18]	2004	Zew Zealand	847	13.6	NR
Lee et al. [19]	2006	USA	709	22.1	4.8
Krishnamurthy et al. [20]	2007	India	1044	8.3	5.5
Simsek et al. [21]	2007	Turkey	158	3.8	5.7
Tubbs et al. [3]	2007	USA	60	5.0	NR
Hong et al. [22]	2008	South Korea	1013	6.5	9.1
Sharma et al. [23]	2010	India	858	4.3	NR
Baeesa et al. [24]	2012	Saudi Arabia	453	16.1	31.8
Bayrakdar et al. [25]	2014	Turkey	730	9.5	11.1
Geist et al. [26]	2014	Taiwan	576	10.4	15.8
Mudit et al. [27]	2014	India	650	2.9	8.0
Chavez and Perez [28]	2015	Peru	1219	8.4	11.1
Chen et al. [29]	2015	Taiwan	500	4.6	2.8
Stropus et al. [30]	2015	Lithuania	706	7.5	24.9
Gibeli et al. [31]	2016	Italy	221	7.7	9.0
Natsis et al. [32]	2019	Greece	244	15.98	18.03
Present study	2025	Greece	300	11.2	18.5

## Data Availability

All the data are available upon reasonable request from the corresponding author (Professor Maria Piagkou, email: mapian@med.uoa.gr).
